# Characteristics of masticatory behavior of patients with mandibular prognathism

**DOI:** 10.1186/s40902-025-00458-9

**Published:** 2025-03-04

**Authors:** Shumpei Mimura, Kanako Kurihara-Okawa, Naoya Fukamachi, Tsukasa Nagasaki, Kazuhiro Hori, Jumpei Okawa, Masaki Takeyama, Takahiro Ono, Isao Saito

**Affiliations:** 1 Division of Orthodontics, Faculty of Dentistry & Graduate School of Medical and Dental Sciences, Niigata University, Niigata, Japan; 2 Division of Comprehensive Prosthodontics, Faculty of Dentistry & Graduate School of Medical and Dental Sciences, Niigata University, Niigata, Japan; 3 Takeyama Orthodontic Clinic, Niigata, Japan; 4 Department of Geriatric Dentistry, Osaka Dental University, Osaka, Japan

**Keywords:** Masticatory behavior, Mandibular prognathism, Wearable device

## Abstract

**Background:**

Patients with mandibular prognathism exhibit not only its characteristic maxillofacial morphology but also discrepancies in stomatognathic function, and understanding this morphology and function is vital for establishing a plan for surgical orthodontic treatment or providing guidance for recovery after orthognathic surgery. However, few studies have yet addressed the objective evaluation of masticatory function before and after surgical orthodontic treatment. In particular, the masticatory behaviors that show how you chew in your daily meals, including the number of chews, chewing rate, and posture during chewing, has not yet been fully studied in patients with mandibular prognathism. The aim of this study was to compare the masticatory behaviors of patients with mandibular prognathism with that of patients with individualized normal occlusion, to clarify the characteristics of masticatory behaviors in mandibular prognathism and to search for a relationship with maxillofacial morphology.

**Methods:**

Participants were 23 patients (12 men, 11 women; mean age 17.9 years) with mandibular prognathism (patient group) and 23 patients (12 men, 11 women; mean age 24.4 years) with normal occlusion that had been achieved by orthodontic treatment (control group). Masticatory behaviors were measured by a wearable device fitted to each participant’s right ear. Parameters such as number of chews, chewing rate, number of chews per bite, mealtime, and head and neck posture were recorded, while participants consumed a 100-g rice ball. Body mass index, occlusal contact area, and lateral cephalograms were also measured, and their associations with masticatory behaviors were investigated.

**Results:**

In patient group, the number of chews was lower, mealtime was shorter, and the head and neck were tilted further forward. There was a significant positive correlation between overjet and anteroposterior head and neck posture and a significant negative correlation between overbite and anteroposterior head and neck posture.

**Conclusion:**

Due to morpho/functional discrepancies in the stomatognathic system, patient group chewed fewer times and for a shorter time and leaned further forward while chewing. The characteristics of the masticatory behaviors of patients with mandibular prognathism identified in the present study may be helpful when devising plans for changing behavior before and after orthodontic treatment or orthognathic surgery.

## Background

Patients with mandibular prognathism exhibit not only its characteristic maxillofacial morphology but also discrepancies in stomatognathic function, and understanding this morphology and function is vital for establishing a plan for surgical orthodontic treatment or providing guidance for recovery after orthognathic surgery. As one aspect of stomatognathic function, masticatory function may be involved in discrepancies in the occlusal relationship, jaw deformity, and the development of temporomandibular arthrosis [1–3], and it is, therefore, important to understand its characteristics. However, few studies have as yet addressed the objective evaluation of masticatory function before and after surgical orthodontic treatment. In particular, the masticatory behavior of patients with mandibular prognathism has yet to be fully studied in terms of the mean number of chews, chewing rate, posture while chewing, and number of chews per bite, parameters that express how much food is chewed and how it is chewed in daily meals. Previous studies of masticatory behavior have used specialized devices with magnets [4] or light-emitting diodes (LEDs) [5] to measure jaw movements, but because these devices must be fitted to the teeth and connected directly to a cable, the possibility that they may obstruct jaw movements cannot be excluded. Observational studies using a video camera have also been conducted; however, because visual evaluations of the movements of the upper and lower jaw are not objective, it is necessary to conduct investigations of intra-investigator measurement error [6]. Basic data on chewing movements when eating daily meals are generally dependent on questionnaires and other subjective data sources, and few quantitative evaluations have been conducted to date.

The bitescan is a wearable mastication measurement device that measures changes in the shape of the back of the ear pinna during jaw movements. It is capable of measuring multiple parameters of masticatory behavior simultaneously without interfering with movements of the lower jaw or neck [7, 8]. It can also wirelessly connect to a smartphone enabling the immediate visualization of masticatory behavior in terms of numerical values in a mobile app. Measuring the masticatory behavior of patients with mandibular prognathism with this device should thus enable the objective assessment of function before and after orthodontic treatment or orthognathic surgery.

This study aimed to investigate the characteristics of mandibular prognathism patient through a comparison with individual normal occlusion patients.

## Subjects and methods

### Participants

The study participants were 23 patients (12 men and 11 women; mean age 17.9 years) diagnosed with mandibular prognathism in the Orthodontics Department at Niigata University Medical and Dental Hospital (patient group). The inclusion criteria comprised the following: the absence of cleft lip and palate or other congenital abnormality; overbite ≥ 0 mm in the incisal region; and on frontal cephalometric radiography at the time of initial examination, deviation of the midline of the mentum (Me) ≤ 5 mm from a vertical midline reference line passing through the crista galli from a horizontal reference line joining the intersections of the inner margins of the left and right orbits with the oblique line (Lo, − Lo′). This was because severe facial asymmetry or lateral deviation of the mandible is reported to cause asymmetric mandibular movement and muscle activity [9, 10], which is likely to give rise to left–right differences in masticatory function. When prognathism is associated with anterior open bite, it is difficult for patients to close their mouth in front, and they are thus unable to carry out the actions required to ingest the test food smoothly, making measurement error likely. Patients with an overbite ≥ 0 mm were therefore included. The control group (control group) comprised 23 patients (12 men and 11 women, mean age 24.4 years) with individual normal occlusion who had completed dynamic treatment in our department and who did not exhibit any morphological or functional abnormality in the maxillofacial region.

The number of subjects was decided by using G*Power 3.1 to calculate the sample size based on data obtained for patient and control groups in preliminary experiments (*α* = 0.05, power = 0.8), which showed that a sample size of 22 patients in each of the patient and control groups was required. In the present study, each group included 23 patients, exceeding the sample sizes calculated with these conditions.

This study was approved by the Ethics Committee of Niigata University (approval no. 2021–0204). All study protocols were conducted in accordance with the Declaration of Helsinki, and informed consent was obtained from all study participants prior to the start of measurements.

### Methods

Various measurements were conducted at the time of initial examination for the patient group and during a hospital visit after the end of dynamic treatment for the control group.

#### Analysis of lateral cephalometric radiographs

**Fig. 1:**
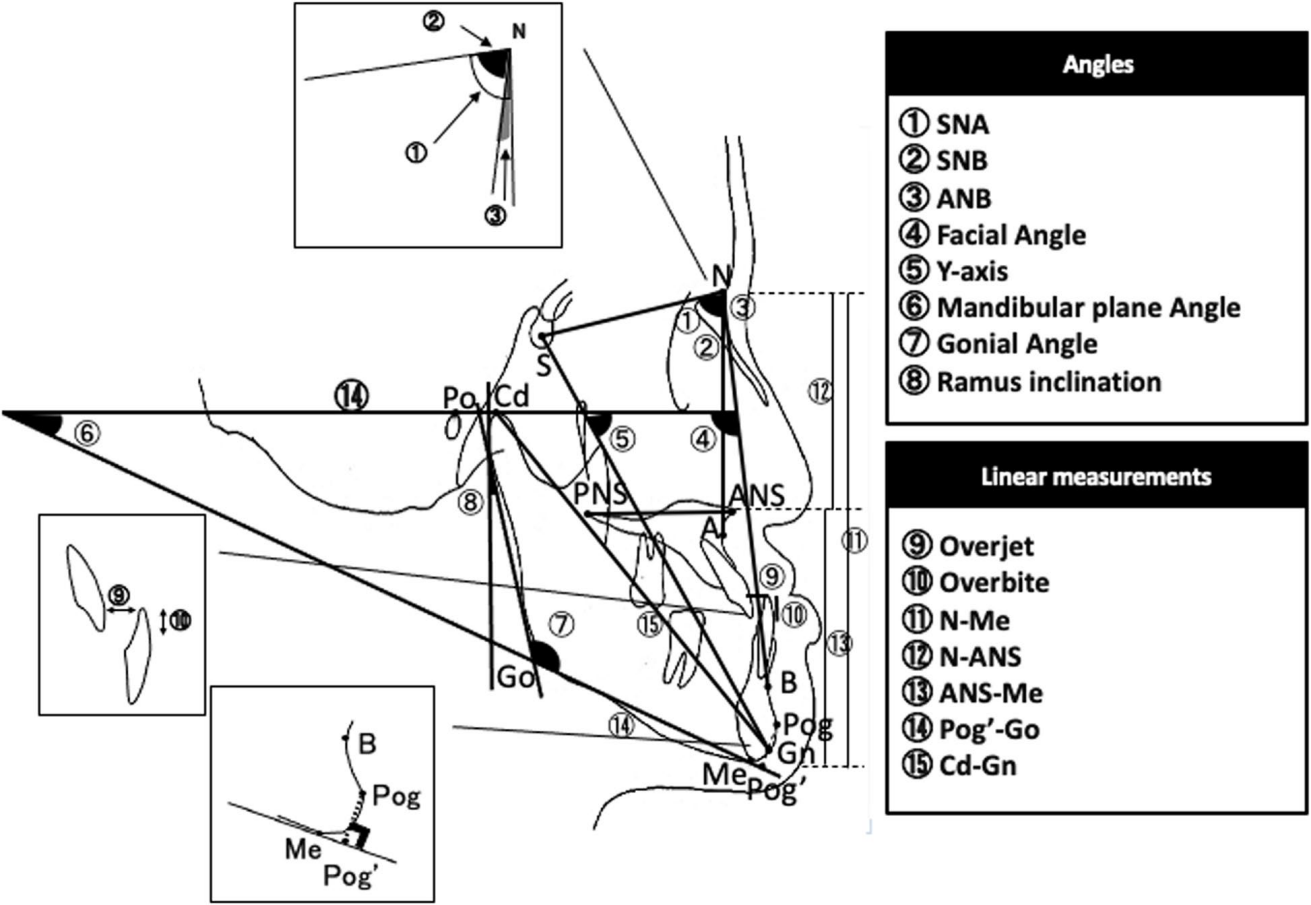
Reference points and planes for cephalometric measurements

Tracings were made of lateral cephalometric radiographs taken at the time of initial examination for the patient group and during a hospital visit after the end of dynamic treatment for the control group, eight angle measurements (SNA, SNB, ANB, facial angle, *Y*-axis, mandibular plane angle, gonial angle, and ramus inclination) and seven distance measurements [anterior facial height (N-Me), upper anterior facial height (N-ANS), lower anterior face height (ANS-Me) (N, ANS, and Me being points scanned in the plain perpendicular to the FH plane), mandibular body length (Pog′-Go) (Pog′ being the point at the foot of a line dropped perpendicularly from Pog in the mandibular plane), mandibular length (Cd-Gn) (Cd being the posterosuperior most point of the mandibular condyle), overjet, and overbite] were made, and their means and standard deviations were calculated (Fig. 1).

#### Body mass index

Height and weight were measured, and the body mass index (BMI) was calculated.

#### Masticatory behavior measurements

**Fig. 2:**
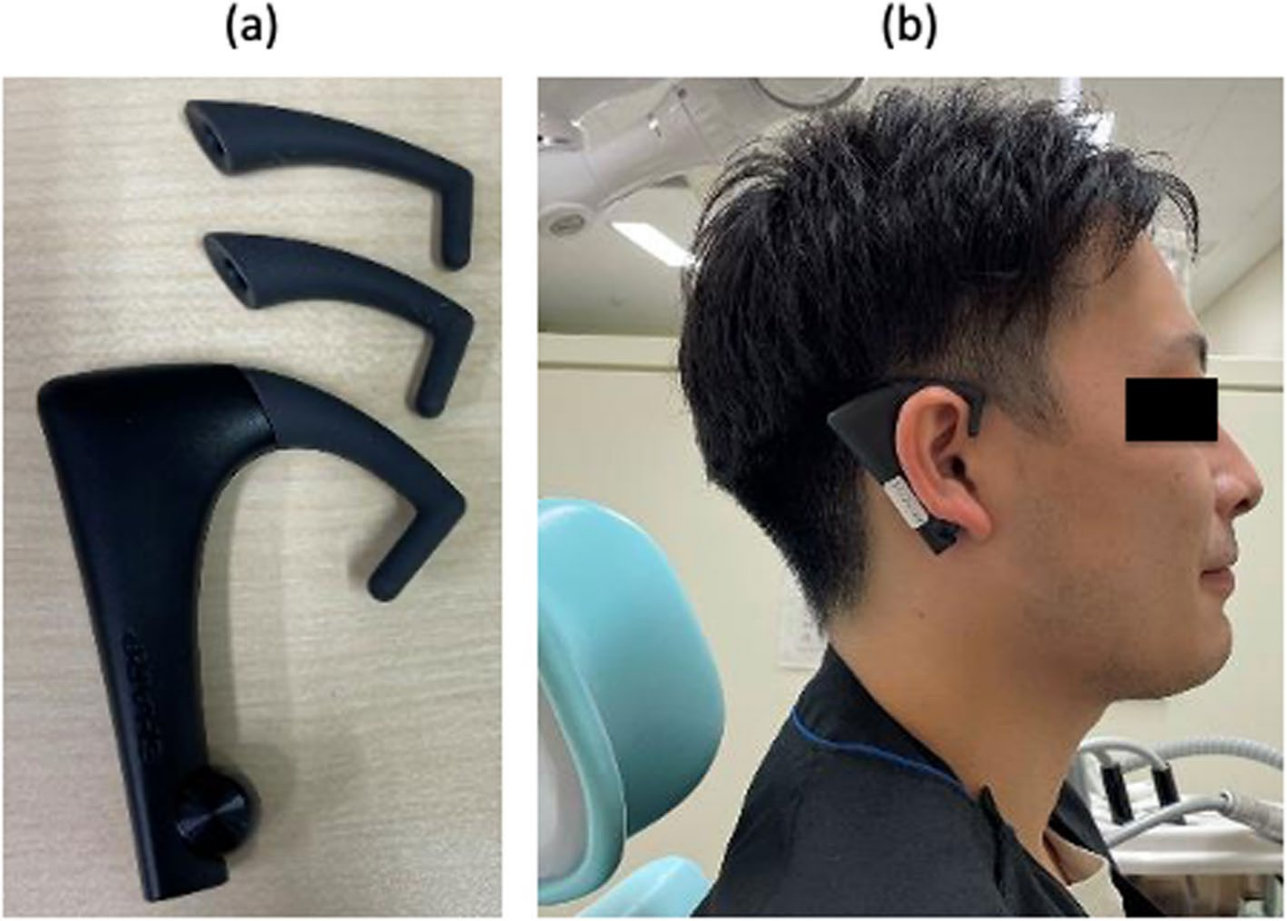
**a** Measurement device for masticatory behavior (Bitescan®). **b** Head and neck posture of the participant during measurement of masticatory behavior

Chewing movements were monitored with a bitescan wearable mastication measurement device (BH-BS1RR, Sharp, Osaka, Japan) (Fig. 2a, b). The device was connected to a smartphone (SH-M15, Sharp) via Bluetooth, and data were collected with a dedicated app (Version 1.7.04). The bitescan is hung over the ear and is available in three sizes (small, medium, and large), with the most appropriate size chosen for each participant. Because the bitescan is designed to be worn on the right ear, it was worn on this side irrespective of the deviated/undeviated side. The device was then calibrated with the participant sitting in a dental chair so that the Frankfort horizontal plane was parallel to the floor. The participant then consumed a 100-g rice ball [norimaki onigiri (kombu), Food Sunaga, Tochigi, Japan] in a natural head position. Each participant ate one rice ball, during which time the number of chews, chewing rate (chews/minute), number of chews per bite, mealtime (seconds), and head and neck posture were recorded.

#### Occlusal contact area measurement

**Fig. 3:**
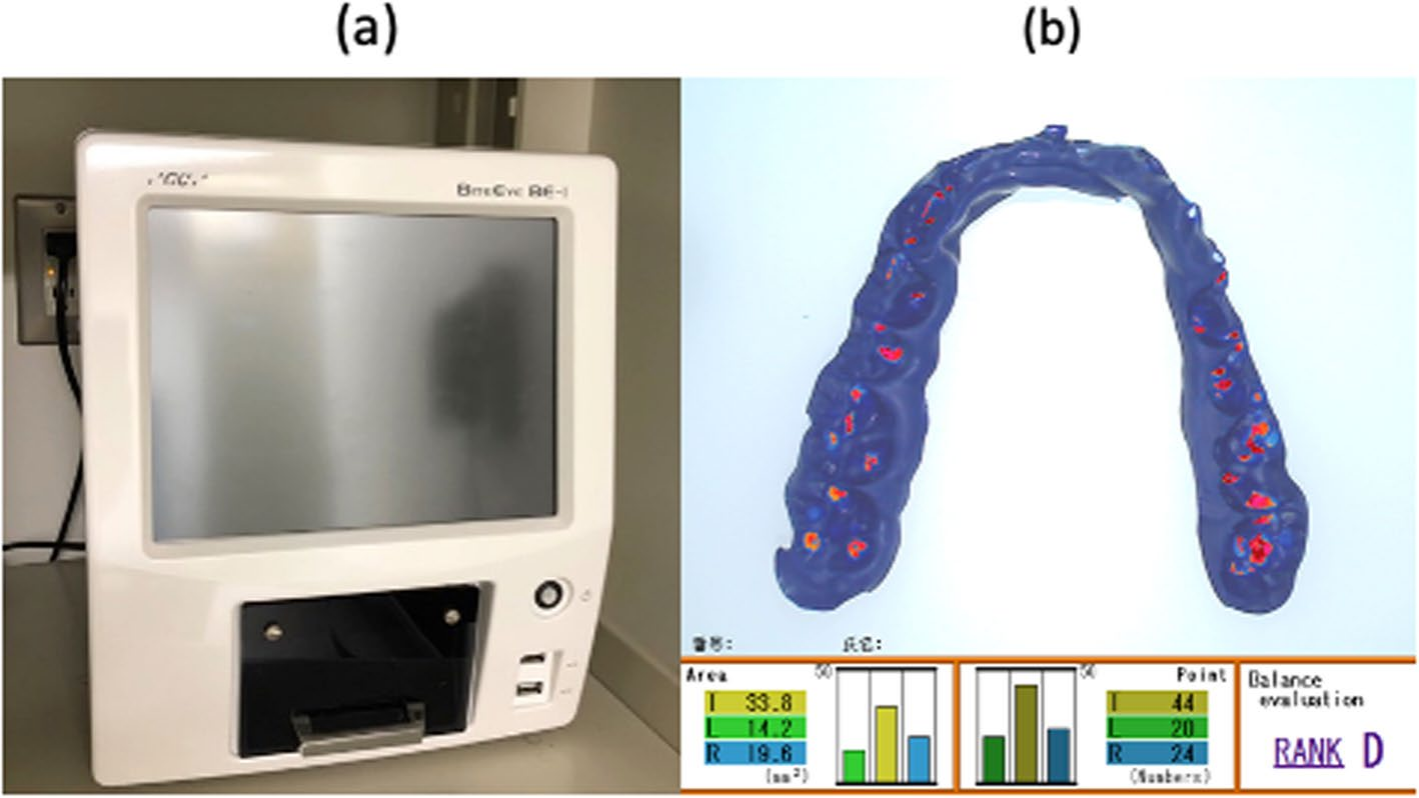
**a** Measurement device for occlusal contact area (Bite Eye®). **b** Magnified screen of Bite Eye®

The participant was seated in a dental chair so that the Frankfort horizontal plane was parallel to the floor, and their maximum intercuspal position was confirmed. Blue silicone material (Blue Silicone, GC, Tokyo, Japan) (Fig. 3a) was then used to record the state of occlusal contact. During blue silicone recording, the participants were instructed to bite down lightly so as to maintain upper and lower occlusal contact for 90 s. The silicone bite recording occlusal contact status was analysed with a tooth contact analyser (Bite Eye, GC) (Fig. 3b), and the occlusal contact area was measured, with sites where the silicone bite thickness was 0–50 μm classed as areas of occlusal contact.

### Statistical analysis

An unpaired *t*-test was used for comparisons of lateral cephalometric radiographic measurements between the patient and control groups, and a Mann–Whitney *U*-test was used for comparisons of the number of chews, chewing rate (chews/minute), number of chews per bite, mealtime, posture, BMI, and occlusal contact area between the two groups.

Spearman’s rank correlation coefficient was used to investigate correlations between lateral cephalometric radiographic measurements and the number of chews, chewing rate (chews/minute), number of chews per bite, mealtime, posture, BMI, and occlusal contact area in the patient and control groups. The statistical software used was IBM SPSS Statistics Version 28 (IBM, Chicago, IL, USA), and *p* < 0.05 was regarded as significant in all cases.

## Results

### Analysis of lateral cephalometric radiographs

In terms of angle measurements, the SNB, Fa, and ramus inclination angles were significantly greater in the patient group than in the control group, and the ANB and *Y*-axis were significantly smaller (Table 1). In terms of distance measurements, the overjet was significantly smaller, and Pog′-Go and Cd-Gn were significantly longer.

**Table 1:** Comparison of mean values of cephalometric analysis in the patient and control groups

	Patients		Control		*p*-value
	*n* = 23		*n* = 23		
	Mean	SD	Mean	SD	
SNA	81.6	4.3	81.2	3.6	0.781
SNB	85.7	4.9	77.6	3.1	< .001
ANB	− 4.2	2.2	3.7	2.2	< .001
Fa	93.3	3.0	86.3	3.0	< .001
Mp	26.2	4.6	28.3	6.1	0.196
Go	126.5	5.6	122.7	7.2	0.052
*Y*-axis	59.6	3.2	63.9	3.3	< .001
Ramus inclination	9.5	4.7	4.4	5.2	0.001
Overjet	− 2.8	2.1	2.5	0.5	< .001
Overbite	2.0	2.1	2.5	0.5	0.324
N-Me	133.8	8.7	131.6	7.3	0.352
N-ANS	59.4	3.9	59.2	3.7	0.847
ANS-Me	74.4	6.7	72.4	5.6	0.288
Pog′-Go	86	6.6	80.0	5.7	0.002
Cd-Gn	135.2	10	124.3	7.5	< .001

*SNA*, SNA angle; *SNB*, SNB angle; *ANB*, ANB angle; *Fa*, facial angle; *Mp*, mandibular plane angle; *Go*, gonial angle; *Y*-axis, *Y*-axis angle

### Comparison of masticatory behaviors, body mass index, and occlusal contact area between the patient and control groups (Fig. 4)

**Fig. 4:**
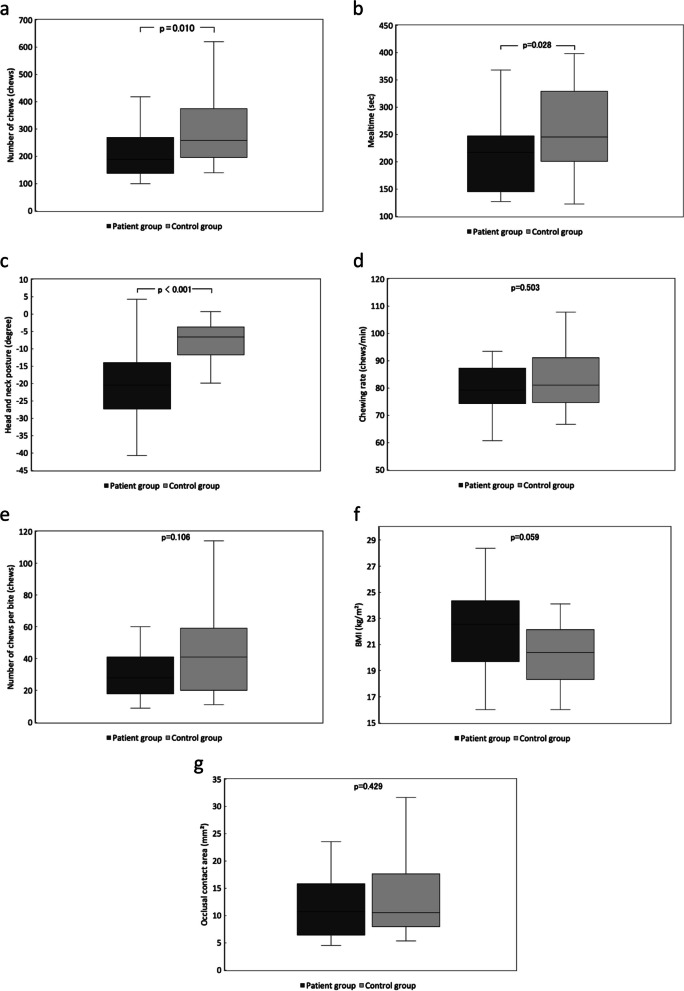
Comparisons of number of chews (a), mealtime (b), head and neck posture (c), chewing rate (d), number of chews per bite (e), BMI (f), and occlusal contact area (g) in the patient and the control group. Box-and-whisker plots indicate the median (line within in box), interquartile range (upper and lower borders of box), and minimum and maximum values (error bars)

The median number of chews was 258 in the control group and 189 in the patient group, significantly fewer in the patient group (*p* = 0.010, Fig. 4a). The median mealtime was 245 s in the control group and 217 s in the patient group, significantly shorter in the patient group (*p* = 0.028, Fig. 4b). In the bitescan assessment of posture, in which a lower number indicates leaning further forward, the median value for leaning backward or forward was − 6.6° in the control group and − 20.5° in the patient group, significantly lower in the patient group (*p* < 0.001, Fig. 4c), who thus leaned further forward while eating. No other significant differences were observed between the two groups. The mean chewing rate was 81.0 chews/min in the control group and 79.3 chews/min in the patient group (*p* = 0.503, Fig. 4d), and the number of chews per bite was 41 chews in the control group and 28 chews in the patient group (*p* = 0.106, Fig. 4e), BMI was 20.4 kg/m^2^ in the control group and 22.5 kg/m^2^ in the patient group (*p* = 0.059, Fig. 4f), and the occlusal contact area was 10.5 mm^2^ in the control group and 10.7 mm^2^ in the patient group (*p* = 0.429, Fig. 4g).

### Association between maxillofacial morphology and masticatory behaviors, body mass index, and occlusal contact area in the patient and control groups (Tables 2 and 3)

**Table 2:** Relationships of maxillofacial analysis items with masticatory behaviors, body mass index, and occlusal contact area in the patient group

Cephalometric analysis	Number of chews	Mealtime	Head and neck posture	Chewing rate	Number of chews per bite	BMI	Occlusal contact area
SNA	− 0.21	− 0.07	− 0.21	− 0.08	− 0.01	− 0.05	0.50*
SNB	− 0.04	− 0.06	− 0.16	0.25	0.31	− 0.03	0.37
ANB	− 0.34	− 0.15	0.03	− 0.42*	− 0.46*	0.13	0.12
Fa	0.02	0.04	− 0.10	0.08	0.09	− 0.09	0.02
*Y*-axis	− 0.03	0.02	− 0.003	− 0.03	− 0.04	0.13	0.10
Mp	0.07	− 0.01	0.36	− 0.18	− 0.06	− 0.11	− 0.19
Go	− 0.19	− 0.10	0.18	− 0.15	− 0.15	0.23	− 0.37
Ramus inclination	− 0.40	− 0.23	0.06	− 0.15	− 0.22	0.40	− 0.14
Overjet	− 0.04	− 0.08	0.46*	− 0.13	0.01	− 0.28	− 0.25
Overbite	− 0.06	− 0.15	− 0.61**	0.22	0.12	0.03	0.32
N-Me	− 0.43*	− 0.39	0.06	0.24	0.07	0.41	0.09
N-ANS	− 0.46*	− 0.54**	− 0.01	0.28	0.15	0.26	0.13
ANS-Me	− 0.35	− 0.14	0.11	0.05	− 0.11	0.41	0.03
Pog′-Go	0.08	− 0.17	0.03	0.46*	0.50*	− 0.14	0.19
Cd-Gn	− 0.24	− 0.29	0.01	0.41	0.28	0.21	0.19

Values in the table indicate correlation coefficients

*SNA*, SNA angle, *SNB*, SNB angle; *ANB*, ANB angle; *Fa*, facial angle, *Mp* Mandibular plane angle, *Go* Gonial angle, *Y*-axis, *Y*-axis angle, *N-Me* anterior facial height, *N-ANS* upper anterior facial height, *ANS-Me*, lower anterior face height, *Pog′-Go* mandibular body length, *Cd-Gn* mandibular length

*p < 0.05

**p < 0.01

**Table 3:** Relationships of maxillofacial analysis items with masticatory behaviors, body mass index, and occlusal contact area in the control group

Cephalometric analysis	Number of chews	Mealtime	Head and neck posture	Chewing rate	Number of chews per bite	BMI	Occlusal contact area
SNA	0.01	− 0.10	0.15	0.35	0.15	0.25	0.24
SNB	− 0.09	− 0.21	− 0.10	0.28	0.06	0.23	0.29
ANB	0.06	0.08	0.35	0.09	0.01	0.16	− 0.02
Fa	0.14	− 0.06	− 0.13	0.39	0.29	0.03	0.27
*Y*-axis	− 0.10	0.08	0.11	− 0.34	− 0.28	− 0.08	− 0.43*
Mp	0.07	0.33	0.21	− 0.39	− 0.46*	− 0.13	− 0.23
Go	− 0.08	0.18	− 0.17	− 0.34	− 0.47*	− 0.15	− 0.17
Ramus inclination	− 0.28	− 0.29	− 0.32	0.02	− 0.15	0.25	0.06
Overjet	− 0.13	− 0.13	0.32	− 0.14	− 0.27	− 0.18	− 0.34
Overbite	0.13	0.04	0.22	0.27	0.19	− 0.02	0.07
N-Me	− 0.54**	− 0.52*	− 0.20	− 0.33	− 0.42*	0.14	− 0.36
N-ANS	− 0.49*	− 0.51*	− 0.28	− 0.14	− 0.16	0.15	0.09
ANS-Me	− 0.35	− 0.33	− 0.02	− 0.29	− 0.40	0.08	− 0.65**
Pog′-Go	− 0.20	− 0.32	0.01	0.04	0.02	− 0.04	0.15
Cd-Gn	− 0.42*	− 0.52*	− 0.30	− 0.05	− 0.15	0.11	− 0.10

Values in the table indicate correlation coefficients

*SNA*, SNA angle; *SNB*, SNB angle; *ANB*, ANB angle; *Fa*, facial angle; *Mp*, mandibular plane angle; *Go*, gonial angle; *Y*-axis, *Y*-axis angle; *N-Me*, anterior facial height; *N-ANS*, upper anterior facial height; *ANS-Me*, lower anterior face height; *Pog′-Go*, mandibular body length; *Cd-Gn*, mandibular length

*p < 0.05

**p < 0.01

**Fig. 5:**
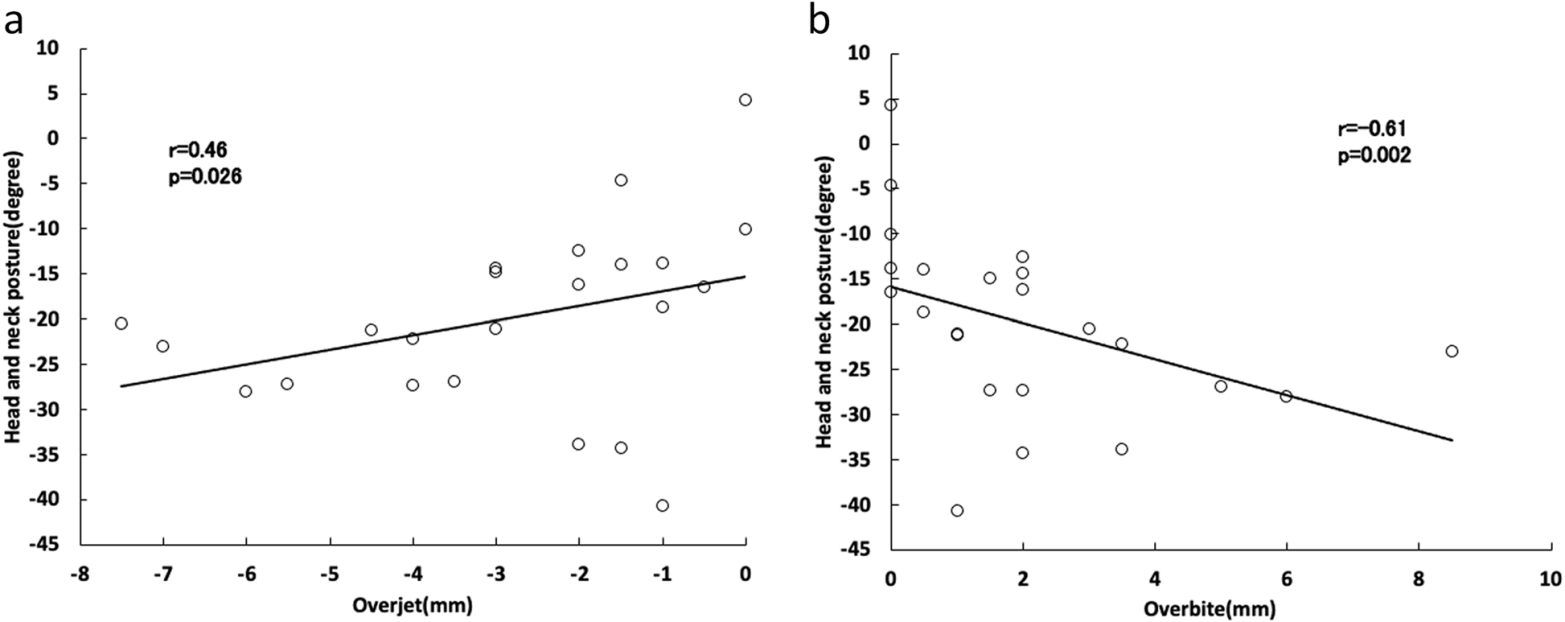
**a** Relationship between overjet and anteroposterior head and neck posture in the patient group. **b** Relationship between overbite and anteroposterior head and neck posture in the patient group

In the patient group, significant positive correlations were observed between the chewing rate and Pog′-Go, between the number of chews per bite and Pog′-Go, and between occlusal contact area and SNA. Significant negative correlations were observed between the chewing rate and ANB, between the number of chews per bite and ANB, between mealtime and N-ANS, and between the number of chews and N-Me and N-ANS. With respect to postural measurements, there a significant positive correlation was observed between overjet and anteroposterior head and neck posture (Fig. 5a), and a significant negative correlation was observed between overbite and anteroposterior head and neck posture (Fig. 5b).

In the control group, significant negative correlations were observed between the number of chews per bite and Mp, Go, and N-Me; between occlusal contact area and *Y*-axis and ANS-Me; between mealtime and N-Me, N-ANS, and Cd-Gn; and between number of chews and N-Me, N-ANS, and Cd-Gn.

## Discussion

This is the first study to conduct a quantitative assessment of the masticatory behaviors of patients with mandibular prognathism using a wearable device. The results showed that compared with participants with individual normal occlusion, patients with mandibular prognathism exhibited characteristic masticatory behaviors comprising a significantly smaller number of chews, significantly shorter mealtime, and leaning further forward while eating. The results also suggested that this masticatory behaviors in prognathic participants may be associated with maxillofacial morphology.

Mandibular prognathism is a misalignment of the positional relationship between the upper and lower dental arches causing a cross bite between the upper and lower front teeth. Either the maxilla is set too far back, the mandible is set too far forward, or both, meaning that the lower jaw projects anteriorly relative to the upper jaw, and, morphologically, this condition is characterized by a long mandible and a large gonial angle [11, 12]. The participants in the patient group in the present study exhibited the characteristic maxillofacial morphology of patients with mandibular prognathism, with mandibles that were located more anteriorly relative to the cranium than those in the control group, as well as a longer mandibular length and longer mandibular body length.

Among other indices, it has previously been reported that patients with mandibular prognathism have significantly lower masticatory performance, occlusal contact area, and maximum occlusal force than do participants with individual normal occlusion [13]. Patients with mandibular prognathism also find it harder than people with normal occlusion to eat a range of different foods in daily life [14], and participants with low masticatory performance reportedly swallow food as large particles [15], both of which may lead to a lower number of chews and shorter mealtime. The present results thus support those of previous studies, suggesting that patients with mandibular prognathism may not fully masticate food taken into the mouth but swallow it while it is still in large particles. However, there have been reports that the masticatory rhythm of patients with mandibular prognathism was unstable and prolonged [16], and that mastication time tended to be longer than that of participants with individual normal occlusion [13]. Those studies involved experimental systems in which the masticatory rhythm and time were measured when participants were instructed to chew for a specified number of times, rather than by monitoring masticatory behavior, and the different measurement method used is likely to have affected the results. Further studies that investigate parameters including masticatory performance are required in the future, as are comparative studies before and after surgery.

The device used in this study, the bitescan, has been investigated for accuracy in previous studies and is reported to be sufficiently reliable [7]. In addition, the bitescan is fitted with a three-dimensional accelerometer for postural evaluation, and it can measure both the movement and inclination of the head. The present measurements of head and neck posture while eating showed that the forward tilt angle was significantly lower in the patient group than in the control group, indicating that this group leaned forward while eating. Patients with mandibular prognathism reportedly tend to adopt a natural head position with the chin tucked in so that their mandibular prognathism is less obvious, out of a fear of being looked at [17]. In this study, even though bitescan calibration was conducted with the Frankfort horizontal plane parallel to the floor, the participants in the patient group may have been used to adopting a natural head position with their chin tucked in, and this may be why they leaned forward while chewing. One study using lateral cephalometric radiographs found that the resting tongue position of patients with mandibular prognathism was overall low and far from the palate, with the tip of the tongue tending to be on the lingual side of the lower front teeth [18]. The position of the hyoid bone has been reported to vary depending on head and neck posture [19], and patients with mandibular prognathism, who have morphological and functional discrepancies of the stomatognathic system, may change the position of the hyoid by leaning forward while chewing to encourage tongue elevation and make it easier to chew and swallow.

The present investigation of the associations between masticatory behavior and maxillofacial morphology found that in the control group, there were significant negative correlations between the number of chews per bite and Mp, Go, and N-Me. In contrast, in the patient group, the chewing rate was significantly negatively correlated with ANB and significantly positively correlated with Pog′-Go, whereas for the items related to posture, anteroposterior head and neck posture were significantly positively correlated with overjet and significantly negatively correlated with overbite. These findings suggested that in the control group, the “high-angle” participants may have taken fewer chews per bite. In the patient group, participants with a longer mandibular body and a greater anteroposterior discrepancy between the upper and lower jaws had a faster chewing rate (chews per minute), and those with a smaller overjet and larger overbite leaned further forward while eating. Given that the number of chews was significantly smaller and mealtime was significantly shorter for participants in the patient group compared with those in the control group, it can be inferred that the participants in this group, who had large discrepancies between their upper and lower jaws and upper and lower front teeth, may not have chewed their food fully. They may have increased their chewing rate by reducing mealtime and encouraged tongue elevation by leaning further forward to swallow the larger particles formed as a result of insufficient mastication. The relationship between masticatory behavior and maxillofacial morphology thus differed between the normal and patient groups, which suggested that the patient group may have both characteristic masticatory behavior and maxillofacial morphology, and that this masticatory behavior may be linked to their maxillofacial morphology. However, as overjet is a potentially important limiting factor in the results of this study, in the future, we intend to further increase the number of participants and make comparisons.

The test food used in this study was a single standard 100-g rice ball. This was chosen so that eating implements such as chopsticks or a spoon would not affect bite size and because the number of chews used to eat a single rice ball is reportedly weakly correlated with the number of chews when eating daily meals [8]. However, there are limitations to the determination and assessment of a large number of masticatory behavior-related items using just a single food, and further studies to evaluate masticatory behavior using test foods with different physical properties, mass, and forms are required in the future.

Studies of mastication have identified sex differences in mastication ability [20]. Both the patient and control groups compared in the present study therefore had equal sex ratios. A number of points have also been raised with regard to the association between mastication and health, and in children in particular, chewing fewer times has been shown to have adverse effects, including weight gain from overeating [21]. Developmental deficiency of oral function in childhood has also been reported to result in the risk of obesity during adolescence [22]. In the present study, the mean BMI of the patient group tended to be higher than that of the control group, but it is possible that in addition to the masticatory behavior of chewing fewer times and for a shorter time, the younger mean age of this group may also have affected their BMI. Future studies should include a larger number of participants to investigate the effects of age and sex differences on masticatory behavior.

It has previously been reported that the occlusal contact area is significantly smaller in patients with mandibular prognathism than in normal participants [13]. In the present study, however, the occlusal contact area was almost identical in both the patient and control groups, with no significant difference between them. This may have been because unlike that previous study, in the present study, the control group consisted of participants with individual normal occlusion who had completed dynamic treatment, including some who had undergone tooth extraction. Given that masticatory behavior is habitual, the results for masticatory behavior obtained from the control group in this study may have reflected the persistence of characteristics acquired prior to orthodontic treatment. Future comparative studies of participants with no history of orthodontic treatment are therefore required.

In a previous study, individuality normal occlusion without previous orthodontic treatment was selected as the control group [13]. However, when these participants were selected as the control group, it was not possible to take lateral cephalograms due to radiation exposure, and patients who had acquired a unique individual normal occlusion by orthodontic treatment alone were selected as a control group and analysed and compared using the lateral cephalograms taken at the end of orthodontic treatment. Patients with orthodontic treatment alone were selected because the group of patients who had achieved individual normal occlusion by orthognathic surgery may have still had the same functional discrepancies in the stomatognathic system that is characteristic of patients with mandibular prognathism. In addition, participants with less anterior–posterior and horizontal discrepancies in maxillofacial morphology were selected because of the possibility that significant discrepancies in maxillofacial morphology could affect masticatory behavior. Based on the above, it is considered that a correct comparison could be made between patients with mandibular prognathism, which is considered to have a large discordance in maxillofacial morphology and function, and a control group. However, it is unclear whether the control group in this study had the same masticatory behavior as those who were born with individual normal occlusion. Therefore, it is necessary to compare the masticatory behavior before and after orthodontic treatment and to examine whether the correct masticatory behavior was acquired by orthodontic treatment intervention in the future.

Masticatory training is conducted after orthognathic surgery with the goals of long-term postoperative stability of the dentition and occlusion, as well as functional improvement, but objective assessment may be lacking, and many issues concerning the verification of functional improvement remain to be resolved. It would provide good motivation for patients by means of visual feedback on their masticatory behavior. It also has the potential to promote changes in masticatory behavior and contribute to habilitation with the new oral and maxillofacial morphology achieved by orthognathic surgery.

## Conclusion

In this study, a bitescan, a small wearable device, was used to measure various parameters associated with masticatory behaviors in patients with mandibular prognathism, and a comparison with participants with individual normal occlusion was performed. It was found that patients with mandibular prognathism chewed significantly fewer times and for a significantly shorter time than patients with individual normal occlusion, and they leaned further forward while chewing. The characteristics of the masticatory behaviors of patients with mandibular prognathism identified in the present study may be helpful when devising plans for changing behavior before and after orthodontic treatment or orthognathic surgery.

## Data Availability

No datasets were generated or analysed during the current study.

## Abbreviations

SNA: SNA angle
SNB: SNB angle
ANB: ANB angle
Fa: Facial angle
Mp: Mandibular plane angle
Go: Gonial angle
Y-axis: *Y*-Axis angle
N-Me: Anterior facial height
N-ANS: Upper anterior facial height
ANS-Me: Lower anterior facial height
Pog′-Go: Mandibular body length
Cd-Gn: Mandibular length
